# The Ongoing Revolution in Breast Imaging Calls for a Similar Revolution in Breast Pathology

**DOI:** 10.1155/2012/489345

**Published:** 2012-09-30

**Authors:** L. Tabár, P. B. Dean, N. Lindhe, M. Ingvarsson

**Affiliations:** ^1^Department of Mammography, Central Hospital, 79182 Falun, Sweden; ^2^Department of Radiology, University of Turku, 20521 Turku, Finland; ^3^Quality Assurance Group, Section of Early Detection and Prevention, IARC, Lyon 69372, France

## Abstract

Communication between pathologists and radiologists suffers from a lack of common ground: the pathologists examine cells in ultrathin tissue slices having the area of a postage stamp, while the radiologists examine images of an entire organ, but without seeing the cellular details. The current practice of examining breast cancer specimens is analogous to scrutinizing individual pieces of a jigsaw puzzle, without examining all of them and never putting all the pieces into place. The routine use of large section histopathology technique could help to alleviate much of this problem, especially with nonpalpable, screen-detected breast cancers. The study of three-dimensional (3D) images of subgross, thick section pathology specimens by both radiologists and pathologists could greatly assist in the communication of findings.

## 1. Introduction

The introduction of large-scale, population-based mammography screening of asymptomatic women at regular intervals—the prerequisite for a significant decrease in mortality from breast cancer—has added a new dimension to the traditional interaction between pathologists and radiologists. The demands upon the screening radiologists are to find the pathologic lesions, including the 5–7 breast cancers among 1,000 asymptomatic women (high sensitivity) and also to rule out the presence of breast cancer in those women who do not have the disease (high specificity). The radiologists perform these demanding tasks by analyzing images of the structural elements of the breast, while the pathologists are primarily concerned with cellular details not seen by the radiologists. 

## 2. The Need to Narrow the Communication Gap between Radiologists and Pathologists

**Figure 1:**
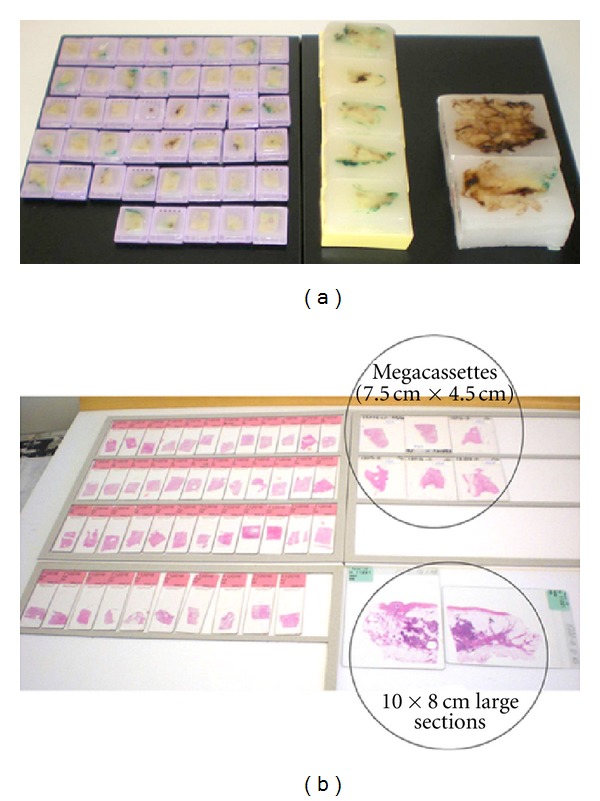
Comparison of the relative number of paraffin blocks (a) and glass slides (b) needed to cover an equivalent area from the same surgical specimen: 45 small, conventional paraffin blocks/slides, five megacassettes/megaslides (7.5 × 4.5 cm), and two 10 × 8 cm paraffin blocks/slides.

The currently used small section histology technique examines samples of the surgically removed breast tissue which are only 2.5 × 2.0 cm in area, while the radiologist images the entire organ. The 4-micron histology slides are viewed at a very high spatial resolution, which reveals cellular details, but the resolution of the breast imaging methods is in the order of 80–100 microns, which is not sufficient to demonstrate cells, but is adequate for visualizing the most important breast structural elements, the ducts, and lobules. To approach a common ground for visualizing breast anatomy and pathology, the small section histopathology technique should be upgraded to the currently available large section (8 × 10 cm) technique [1–4]. The small section histology technique in current use is inadequate for the correlation of imaging findings in most multifocal and diffusely infiltrating breast cancer cases as the piecemeal reconstruction of the tumor foci and surgical margins is unlikely to have a realistic 1 : 1 correlation with modern imaging methods. Large section histology enables the examination of about 65 cm^2^ of contiguous tissue, while the standard glass slides greatly limit the reliable assessment of tumor size and disease extent (Figures 1(a) and 1(b)).

**Figure 2:**
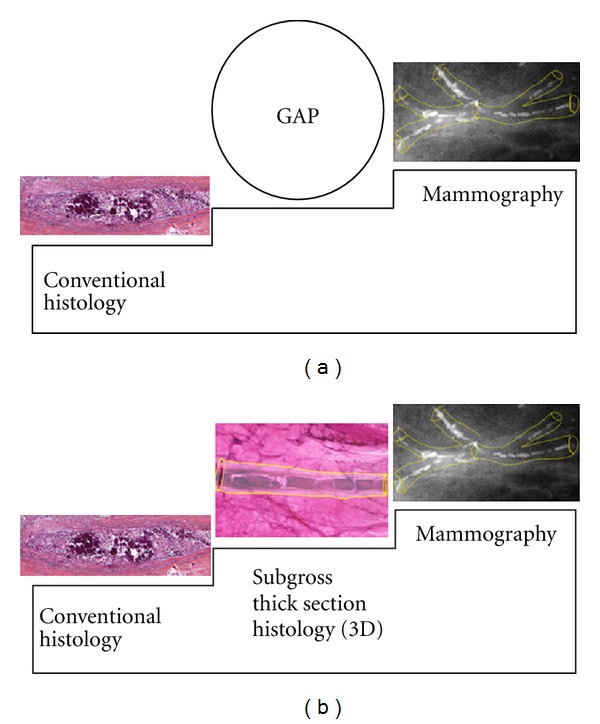
(a) A stepwedge expressing the discrepancy between the low-resolution mammographic image and high-resolution histologic image. The mammogram shows calcifications localized in a well-defined *structure* of the breast (ducts), while the interpretation of the histopathologic finding emphasizes the *cellular details* not seen by the radiologists. There is a communication gap since the radiologist expects a detailed description of tissue structure, in addition to the description of cellular features. (b) The communication gap can be overcome with the help of subgross (3D) histology. In this case the location of fragmented casting type calcifications and the intraductal pathologic process producing them can be clearly seen on the subgross (3D) image, providing excellent radiologic-pathologic correlation.

Whereas the mammogram is a projection image of all the 3-dimensional breast anatomic structures superimposed, the thin (4-micron) section histopathology slides provide a highly detailed 2-dimensional image of a slice through the ducts and lobules. To view these structural elements in their entirety, the histopathology slice needs to be on the order of 1,500 microns, called the large section subgross (3D) histology technique. This technique can serve to bridge the gap that separates the viewpoints of the pathologist and radiologist (Figures 2(a) and 2(b)).

Combining the large thin section (10 × 8 cm) histology technique with the subgross, 3D method facilitates a better understanding of normal and pathologic breast tissue and makes precise correlation with the radiologic imaging methods a reality. “Progress in histologic-mammographic correlation can only be made by examining a histologic specimen of greater length, width and depth” [1]. The current practice of histopathology of breast specimens has serious limitations. “Complete specimen examination is rarely performed in clinical practice” and “In a typical 8-cm diameter lumpectomy specimen, assuming four conventional pathology margin sections are removed in a single plane, only 16% of the circumference is examined microscopically” [5]. In contrast, because the large section histology technique covers a contiguous area which is 10–20 times larger than the small section, it facilitates a more comprehensive evaluation of the lesion(s) relative to the surrounding tissue, a more accurate assessment of the true extent of the disease (including the number of tumor foci) and more complete evaluation of the surgical margin [6]. All these benefits culminate in more accurate diagnoses, leading to more appropriate treatment, fewer recurrences, fewer reoperations, and improved patient care.

**Figure 3:**
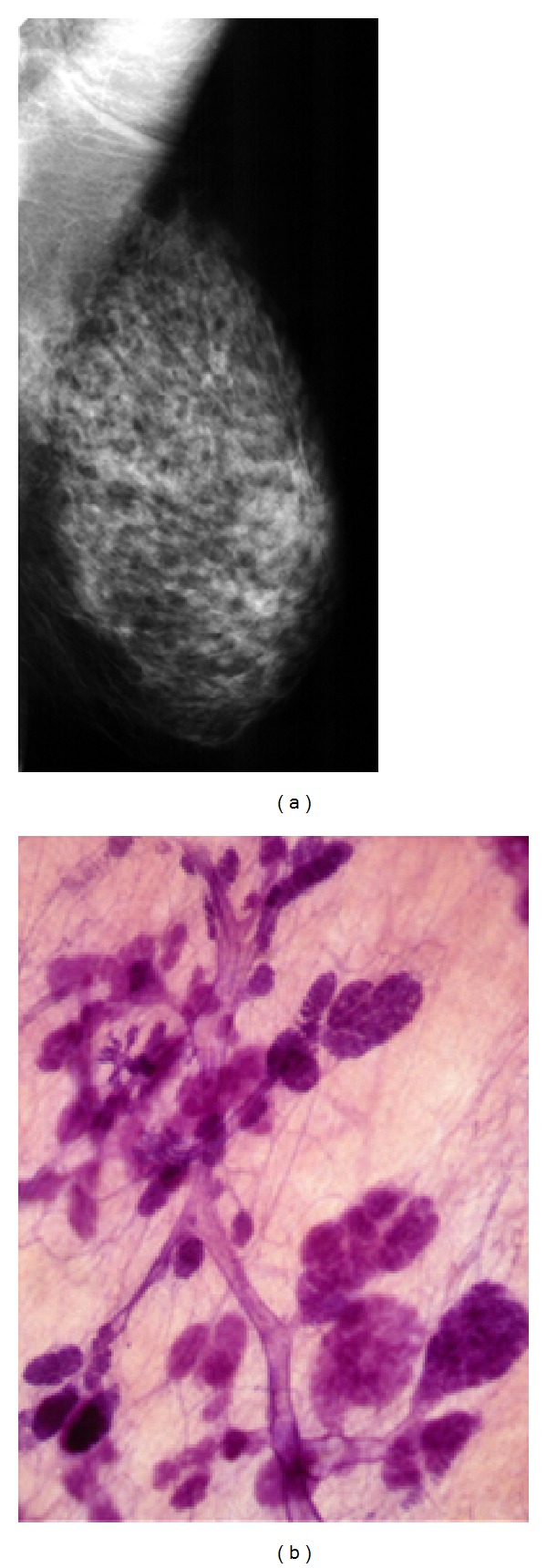
Mammogram of the left breast in the mediolateral oblique projection (a) and large section, subgross (3D) histology of normal breast tissue. (b) On the mammogram the nodular/oval shaped densities (TDLUs) and the linear densities (larger milk ducts) are surrounded by radiolucent adipose tissue, making it possible to account for each nodular and linear density. The nodular densities are the radiologic images of the lobules of varying sizes, and the linear densities are ducts as seen on the 3D histology.

Special training in mammographic-histologic correlation, using the techniques of large thin/thick section histology, can enable the radiologist to account for every detail on the normal mammogram (Figures 3(a) and 3(b)). 

Trained in this manner, the radiologist will be able to maximize the benefits of screening, while minimizing over/underdiagnosis. 

## 3. Establishing the Diagnosis and Determining the Extent of the Disease

**Figure 4:**
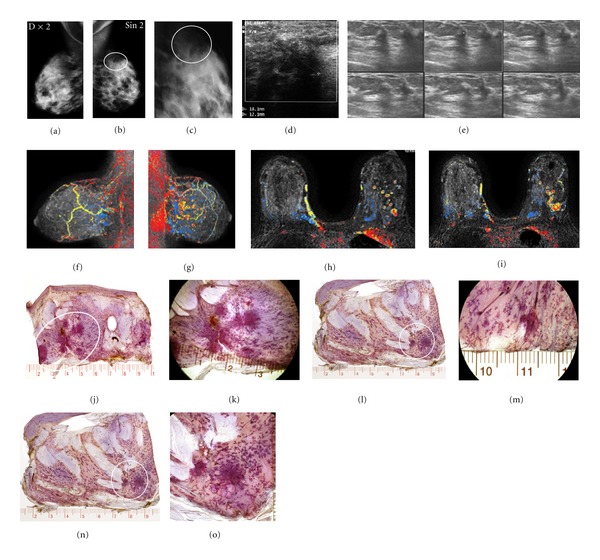
Right (a) and left (b) mammograms, mediolateral oblique projections. Subtle architectural distortion and parenchymal contour change are confirmed on the microfocus mammographic image (c) of the left breast (encircled). Hand-held ultrasound (d) and automated, 3D ultrasound (e) images confirm the presence of malignancy and suggest multifocality. Magnetic resonance examination in the sagittal (f, g) and axial (h, i) planes demonstrates fifteen separate malignant tumor foci. Since only 3 invasive tumor foci were described on the postsurgical histopathology report, the remainder of the mastectomy specimen was also prepared for microscopic examination. Three subgross (3D) histology slices from the reexamination of the mastectomy specimen (j, l, n) and photomicrographic magnification of the areas containing the 12 additional individual invasive cancer foci (k, m, o). This reexamination completes the radiologic-pathologic correlation.

Having found an abnormality on the mammogram, the multimodality approach is used to establish the preoperative diagnosis. This includes a combination of imaging methods and image-guided percutaneous biopsy. Determination of disease extent is an integral part of the preoperative diagnostic workup in order to provide better treatment planning. In particular, it is important to distinguish between unifocal, multifocal, and diffusely infiltrating cancers preoperatively, as the therapeutic approach must necessarily be different for each to avoid under/overtreatment. Determination of disease extent can be accomplished with modern, high-quality imaging tools, in particular preoperative magnetic resonance imaging (MRI) of the breast. Correlating the findings of modern imaging techniques with large section histology on a routine basis will lead to a considerably improved mapping of disease extent, which helps to prevent incomplete resection of breast cancer at primary surgery. Incomplete resection of invasive cancer foci is associated with a poor outcome: “For patients who underwent second surgery, the finding of a residual invasive carcinoma was associated with increased risk for distant recurrence (22.8% versus 6.6%; HR 3.5; 95% confidence interval, 1.8–7.4; *P* < .0001)” [7]. The fatality ratio for multifocal and diffusely infiltrating tumors is 2 and 3 times greater, respectively, compared to unifocal tumors of the same TNM size range [6]. This greater fatality ratio emphasizes the importance of determining the full extent of the disease preoperatively. As breast imaging tools, particularly breast MRI, continue to improve in finding more and smaller tumor foci, radiologists all over the world experience a disturbing scenario, in which there may be a considerable discrepancy between the number of tumor foci and disease extent found at MRI and the corresponding number of tumor foci and disease extent described in the pathology report. In these cases the radiologist may be accused of overdiagnosis. “People blame MRI instead of the limitations of conventional pathology *and* a failureof small sectionpathology to correlate with MRI and mammography” (Lee Tucker, M.D., F.C.A.P., personal communication 2012). The following is a typical example. An asymptomatic 50-year-old woman was called back from screening for assessment of a subtle parenchymal contour change in the axillary tail of the left breast, seen on the mediolateral oblique projection (Figure 4(b)). At preoperative breast MRI fifteen separate tumor foci were identified. Several foci were biopsied under ultrasound guidance. Histology of the 14 g core biopsy specimens confirmed the malignant nature of these foci, and mastectomy was performed.

**Figure 5:**
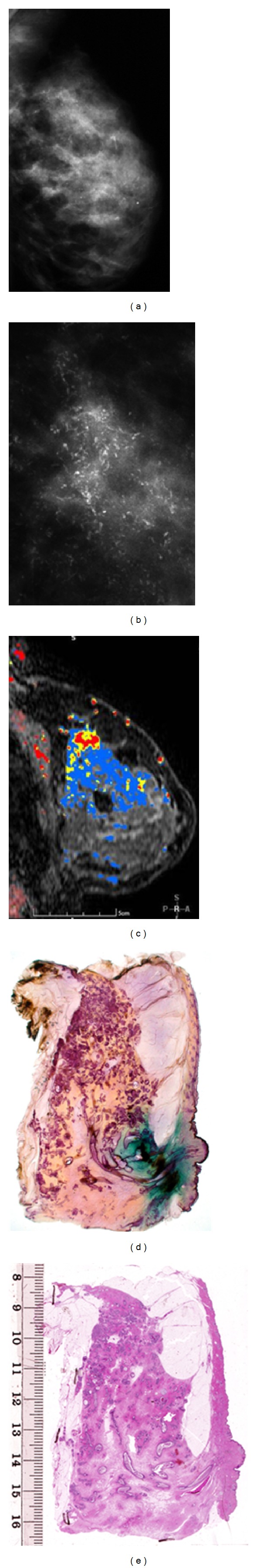
Left breast, mediolateral oblique projection (a) and microfocus magnification (b). Casting type calcifications are seen in the entire upper portion of the left breast, indicating an extensive malignant process within the ducts. The breast MRI examination (c) confirms the extensive nature of the disease and detects an invasive focus in the axillary tail. Large thick section (subgross, 3D) (d) and large thin section (e) histology provide excellent correlation with the imaging findings by demonstrating a 12 × 12 mm invasive breast cancer and a large number of cancer-filled, distended, duct-like structures occupying the entire upper portion of the left breast.

The histology report of the mastectomy specimen described only three invasive tumor foci. As is customary, the pathology report was regarded as the gold standard and the radiologist was accused of overcalling, which, allegedly, led to an unnecessary mastectomy. At the instigation of the radiologist, a thorough examination of the remaining mastectomy specimen was performed, and 12 additional invasive cancer foci were found (Figures 4(j)–4(o)). In this case, as in many other cases, MRI was blamed for overcalling, when in reality, it is the histopathologic examination that was undercalling. In other situations, such as in extensive *in situ* carcinomas or diffusely infiltrating invasive cancers, the specimen should be sliced according to the location and extent of the disease as demonstrated with preoperative imaging (Figures 5(a)–5(e)). 

Exclusive use of the “breadloafing” technique may seriously underestimate the true extent of diffuse breast cancers (diffusely infiltrating classic invasive lobular carcinomas and extensive microcalcification cases), because in the flattened mastectomy specimen the distance between the skin and the chest wall will be minimized. For example, if the mammograms show malignant type calcifications extending from the nipple to the chest wall over a distance of 8 cm, and the breast MRI also shows a similarly large extent of the disease, then one might expect that the same or larger extent of the disease will be found at histopathologic examination. In such cases slicing of the mastectomy specimens should take the geometry of the malignancy into account, which may require slicing the specimen parallel to the table, instead of using the breadloafing approach. Cooperation between the radiologist and pathologist should prevent discrepancies in determination of true disease extent. 

We recommend that *large section histopathology should be standard* for all breast cancer surgical specimens, as it also provides better correlation with breast imaging.
